# A Kramers-Moyal Approach to the Analysis of Third-Order Noise with Applications in Option Valuation

**DOI:** 10.1371/journal.pone.0116752

**Published:** 2015-01-27

**Authors:** Dan M. Popescu, Ovidiu Lipan

**Affiliations:** 1 Department of Physics, University of Richmond, Richmond, Virginia, United States of America; University College London, UNITED KINGDOM

## Abstract

We propose the use of the Kramers-Moyal expansion in the analysis of third-order noise. In particular, we show how the approach can be applied in the theoretical study of option valuation. Despite Pawula’s theorem, which states that a truncated model may exhibit poor statistical properties, we show that for a third-order Kramers-Moyal truncation model of an option’s and its underlier’s price, important properties emerge: (i) the option price can be written in a closed analytical form that involves the Airy function, (ii) the price is a positive function for positive skewness in the distribution, (iii) for negative skewness, the price becomes negative only for price values that are close to zero. Moreover, using third-order noise in option valuation reveals additional properties: (iv) the inconsistencies between two popular option pricing approaches (using a “delta-hedged” portfolio and using an option replicating portfolio) that are otherwise equivalent up to the second moment, (v) the ability to develop a measure *R* of how accurately an option can be replicated by a mixture of the underlying stocks and cash, (vi) further limitations of *second*-order models revealed by introducing *third*-order noise.

## Introduction

Finance is one of the areas in which there exists a strong emphasis on high-order fluctuations, as these can have a massive impact on markets. Arguably, the most common formula in financial mathematics is the Black-Scholes-Merton [1, 2] used in option valuation. Viewing it not from an Itô, but from a Kramers-Moyal (KM) perspective, Black-Scholes-Merton employs a truncation of terms in the KM expansion from infinity down to the second order. It is tempting to truncate the terms down to a higher order such as the third, thereby introducing third-order noise. The existence of a third order enriches the model with a skewness parameter, *γ*, in a natural way that preserves the economic interpretation. However, Pawula’s theorem warns that, once the third order is accounted for, the model may end up exhibiting poor properties. For example, it may turn out that the option price takes negative values for a wide range of stock prices. Or, that the price will be positive only for few selective regions of the skewness parameter. Before studying the truncated model and show that it displays useful properties, we discuss the importance of the stock price stochastic properties in building a model for the option price.

Our aim is not to tweak the Black-Scholes-Merton option pricing formula, but to provide a theoretical framework for working with third-order noise. We chose the Black-Scholes-Merton as a starting point given the widespread focus in the literature regarding the lognormality assumption underlying it. Several attempts were made in the last 40 years to account for non-Gaussian noise in the underlying stock price distribution. One of the first was Merton’s [3] through the introduction of a Poisson jump process in addition to the classic diffusion process. This model captures a variety of stock price movements through three variables—the average number of jumps per unit time and the mean and standard deviation of the jump size. A different approach taken to relax the normality assumption was to consider the volatility parameter as a random variable (see [4–6]). Other models start with a modified normal distribution. For example, Jarrow and Rudd [7] perform an Edgeworth expansion of the lognormal distribution of asset prices, whereas Brigo and Mercurio [8] assume the underlying asset price distribution is a mixture of lognormals. Lastly, we would like to mention local volatility models (see [9–11]), in which the volatility of the underlying asset is a function of time and the underlying asset’s price. An example of how this model can be used is to assume that the Black-Scholes-Merton partial differential equation still holds and to infer the local volatility from the estimated implied volatility smile.

In this paper, we assume that observations are carried out on a time scale small enough to consider the price returns as being well approximated by a Markov process. We then use the Chapman-Kolmogorov equation in its differential form (also known as the Master Equation) to describe movements in price [12]. Under certain conditions [13], the differential Chapman-Kolmogorov equation decomposes the process into drift, diffusion and jumps. The drift and diffusion components are part of the classic lognormal description of asset prices, whereas the jumps were used by Merton in the form of a compound Poisson process. Alternatively, instead of decomposing the Master Equation, we will use a formally equivalent representation, the KM expansion [14, 15]. This provides the benefit of truncation after a suitable number of terms [12]. Although Pawula’s theorem limits the truncation possibilities to second order or to infinity, Risken [16] shows that truncations of the KM expansion are useful and offer good approximations to probability distributions.

## Results

Despite the discouraging possibility that Pawula’s theorem can make the price distribution unintuitive, it turns out that the third-order KM option pricing model has the following useful proprieties: (i) the price can be written in a closed analytical form that involves the Airy function, (ii) the price is a positive function for any *γ* > 0, (iii) for *γ* < 0, the price becomes negative only for price values that are close to zero, (iv) the third-order moment distinguishes between two popular derivation methods of the Black-Scholes-Merton equation, which are otherwise equivalent up to the second moment, (v) from the discrepancy between the two methods, it is possible to infer a measure *R* of how precisely options can be replicated using the underliers and cash, (vi) limitations of models truncated from infinity to the second order are revealed.

For example, to point out property (i), the cost of the option with expiration time *T* and strike *K* is

$$c(s,t)=\pm e^{-r\tau }Ke^{-\frac{1}{3}{\left(\eta -\beta \right)}^{3}}\int_{a}^{\pm \infty }d\xi \left(e^{-\beta a}-e^{-\beta \xi }\right)e^{\eta \xi }\text{Ai}(\xi ),$$ (1)

with ± = sign(*γ*), *η* = (*σ*/*γ*)^2^(*τ*/2)^1/3^, *β* = *γ*(*τ*/2)^1/3^, *a* = (*m* ^2^/3*n*)(*τ*/*β*) − *x*/*β*. Here, *m* = 2^−1^(*σ* ^2^ − *γ* ^3^), *n* = 6^−1^ *γ* ^3^ and

$$x\;=\text{ln}\left(\frac{s}{K}\right)+\left(r-\frac{1}{2}\sigma^{2}+\frac{1}{3}\gamma^{3}\right)\tau ,$$ (2a)

τ=T−t. (2b)

The asymptotic expansion of the cost for small values of *γ* makes the Black-Scholes-Merton formula apparent in the limit *γ* → 0

$$c(s,t)\;=\;s\frac{1}{2\pi }\int_{-\infty }^{d_{+}}d\theta \left(1+\gamma^{3}\Omega \left(\theta -\sqrt{(\sigma^{2}-\gamma^{3})\tau }\right)\right)e^{-\theta^{2}/2}\;-\;Ke^{-r(T-t)}\frac{1}{2\pi }\int_{-\infty }^{d_{-}}d\theta \left(1+\gamma^{3}\Omega (\theta )\right)e^{-\theta^{2}/2},$$ (3)

where

$$d_{\pm }=\frac{\text{ln}\left(\frac{s}{K}\right)+\left(r\pm \frac{1}{2}\left(\sigma^{2}-\gamma^{3}\right)\right)\tau }{\sqrt{(\sigma^{2}-\gamma^{3})\tau }},$$ (4)

and the correction term

$$\Omega (\theta )=\frac{1}{4}\frac{\theta }{\sqrt{(\sigma^{2}-\gamma^{3})\tau }}\left(1-\frac{\theta^{2}}{3}\right).$$ (5)

The effect of the new skewness parameter, *γ*, is that the stock price stochastic process is no longer lognormal, as it is in the Black-Scholes-Merton model.

## Model

The stochastic process for each financial instrument is described by a corresponding probability density *p*(*z*, *t*), where *z* is the value of the financial instrument at time *t*. The probability density is treated as an unknown that satisfies the KM equation:

$$\frac{\partial p(z,t)}{\partial t}=\sum_{n=1}^{\infty }{\left(-\frac{\partial }{\partial z}\right)}^{n}\left(D^{\left(n\right)}(z,t)p(z,t)\right).$$ (6)

Here, the *D* ^(*n*)^(*z*), *n* = 1, 2, …, called the *derivate transition moments* for the process [16], are inferred from financial data and are, therefore, considered to be known functions of *z* (for a discussion on their estimation, see [17]). In this manuscript, the cost of the option, *c*(*s*, *t*), is obtained from solving a partial differential equation whose coefficients are completely based on the derivate transition moments of the underlying stocks and risk-free bonds. The reasoning employed to deduce the cost of the option is based on the transition probability moments of the financial instruments. They are given by *M* ^(*n*)^(*z*, *t*, *ϵ*) = *∫* *dZ*(*Z* − *z*)^*n*^ *p*(*Z*, *t* + *ϵ*∣*z*, *t*), where *p*(*Z*, *t* + *ϵ*∣*z*, *t*) is the transition probability for the stochastic process to have the value *Z* at time *t* + *ϵ* given that it had the value *z* at time *t* and integrals without limits are considered from −∞ to ∞. The stochastic transition takes place during a small time interval *ϵ*, between *t* and *t* + *ϵ*. The transition probability moments and the derivate transition moments are connected through $M^{\left(n\right)}(z,t,\epsilon )=\epsilon n!D^{\left(n\right)}(z,t)+O(\epsilon^{2})$.

When considering a truncation of the Kramers-Moyal expansion in (6), it is important to know how many terms of the expansion should be taken into account such that the approximation reaches the desired level of precision. Pawula’s theorem shows that the positivity constraint on the probability, *p*(*z*, *t*) ≥ 0, translates into a constraint on the KM expansion. Namely, the KM expansion must stop either after the first or after the second term. If it does not stop after the second term, then it must contain an infinite number of terms [18]. That is, if the expansion has a non-zero third-order term, then it must contain an infinity of terms.

The theorem, however, does not say that expansions truncated after or at the third order are of no use. If the negative values for *p*(*z*, *t*) resulting from the approximate distribution are small or confined to a small region of *z* and *t*, the resulting approximation could be very good [16]. Furthermore, van Kampen [12] points out that, at least in physical systems, nonzero moments higher than the second order are to be expected. Gardiner [13] also shows the importance of higher-order terms (in particular, the third) in the treatment of trimolecular reactions. This warrants a similar higher-order approach in the case of stock prices as well.

In the development of the model, we will assume ideal conditions similar to those present in the Black-Scholes-Merton model [1]: (a) The markets are “frictionless”: transactions do not incur fees and costs. (b) Participants in the market can borrow and lend cash at the risk-free rate. (c) The underlying security does not pay dividends. (d) The option is “European”, therefore one can exercise it at maturity only. (e) There are no restrictions on borrowing or short-selling. (f) The market is arbitrage-free. (g) The market is “perfectly liquid”: participants can buy or sell any amount (positive or negative) of the asset. It is worth pointing out that assumptions (a)-(d) are relatively weak. Methods have been designed in the literature to relax them. Assumptions (e), (f) and (g) are, however, strictly required.

Several types of financial instruments are used in this paper: (i) *options* represent securities with value *c*(*s*(*t*), *t*) at time *t* written on an underlier *s*(*t*) (in this case, common stock). The analysis will focus on European-style “call” options with maturity *T* and strike price *K*. The option has non-zero higher-order derivate moments $D_{c}^{\left(2\right)}$, $D_{c}^{\left(3\right)}$ …; (ii) *stocks*, with value *s*(*t*) at *t*, are the underliers for the option. The value *s*(*t*) is random with higher-order non-zero derivate moments: $D_{s}^{\left(2\right)}$, $D_{s}^{\left(3\right)}$ …; (iii) *risk-free bonds* with value *b*(*t*) at time *t* are assets earning the risk-free rate. Since they have no fluctuations, $D_{b}^{\left(i\right)}=0$ for *i* ≥ 2.

A *portfolio* of *n* assets *z*_*i*_(*t*) is defined as $\sum_{i=1}^{n}\Delta_{i}(t)z_{i}(t)$, where Δ_*i*_ is the quantity of asset *i* with price *z*_*i*_ per unit. A portfolio is self-financing if its value after a small positive increment in time *ϵ* ∈ [0, Δ*t*), Δ*t* > 0, is given by $\sum_{i=1}^{n}\Delta_{i}(t)z_{i}(t+\epsilon )$, which means that the change in the value of the portfolio during a short time interval Δ*t* comes only from the change of the price of the assets and not from the change in the quantities of assets Δ_*i*_(*t*).

Two popular approaches used to derive the option dynamics in the Black-Scholes-Merton framework are: the *delta-hedged* portfolio method (DHP) and the option replicating portfolio method (ORP). In models based on KM truncations from infinity to the second order, the two methods are equivalent. However, when considering the third order, two noteworthy results emerge: (i) the two methods are no longer perfectly equivalent and (ii) the replication in the ORP is not perfect, so a measure of the accuracy of replication is necessary.

In describing the methods, upper-case letters and lower-case letters will be used to distinguish between each variable at two different times *t* and *t* + *ϵ*, respectively. For example, *c*(*s*(*t* + *ϵ*), *t* + *ϵ*) ≡ *C*, *c*(*s*(*t*), *t*) ≡ *c*.

### Delta-hedged portfolio method

In DHP, the aim is to create a self-financing portfolio $f=-c(s,t)+\Delta_{1}^{\left(f\right)}(t)s$ by going short one unit of the option *c*(*s*, *t*) and long a quantity $\Delta_{1}^{\left(f\right)}(t)$ of *s*(*t*) chosen such that the portfolio is risk-free, that is, it has no fluctuations $M_{f}^{\left(2\right)}=M_{f}^{\left(3\right)}=\ldots =0$. The *no-arbitrage* condition requires that a portfolio with no risk have a return equal to the risk-free rate. That is,

$$M_{f}^{\left(1\right)}=M_{b}^{\left(1\right)},$$ (7)

where *b* is a bond returning the risk-free rate. This equation will be used to find an explicit form for *c*(*s*, *t*) from the moments of *f* as a function of the moments of *s*:

$$M_{f}^{\left(1\right)}=-\frac{\partial c}{\partial t}\epsilon -M_{s}^{\left(1\right)}\left(\frac{\partial c}{\partial s}-\Delta_{1}^{\left(f\right)}(t)\right)-\frac{1}{2}M_{s}^{\left(2\right)}\frac{\partial^{2}c}{\partial s^{2}}-\frac{1}{6}M_{s}^{\left(3\right)}\frac{\partial^{3}c}{\partial s^{3}}+\ldots +O(\epsilon^{2}),$$ (8a)

$$M_{f}^{\left(2\right)}={\left(\frac{\partial c}{\partial s}-\Delta_{1}^{\left(f\right)}(t)\right)}^{2}M_{s}^{\left(2\right)}+\frac{1}{2}\frac{\partial^{2}c}{\partial s^{2}}\left(\frac{\partial c}{\partial s}-\Delta_{1}^{\left(f\right)}(t)\right)M_{s}^{\left(3\right)}+\ldots +O(\epsilon^{2}),$$ (8b)

$$M_{f}^{\left(3\right)}={\left(\frac{\partial c}{\partial s}-\Delta_{1}^{\left(f\right)}(t)\right)}^{3}M_{s}^{\left(3\right)}+\ldots +O(\epsilon^{2}),$$ (8c)

where in (8a), (8b), (8c), the “…” stand for terms containing the moments *M*_*s*_ of order higher than the third. The moments $M_{f}^{\left(i\right)}$ for *i* ≥ 4 depend only on moments *M*_*s*_ of forth and higher order plus terms of order $O(\epsilon^{2})$.

Such a risk-free portfolio can be constructed if the following two conditions are fulfilled. First, the KM expansion for the stock *s*(*t*) is truncated after the third term, i.e. $M_{s}^{\left(4\right)}=M_{s}^{\left(5\right)}=\ldots =0$. Second, the particular choice for the amount of stock held in the portfolio during each interval of time [*t*, *t* + Δ*t*), Δ*t* > 0 is $\Delta_{1}^{\left(f\right)}(t)=\partial c(s,t)/\partial s$.

### Option-replicating portfolio method

In the ORP method, the goal is to create a portfolio of the underlying stocks and risk-free bonds that replicates a capital-equivalent investment in options. To achieve this, form a self-financing portfolio of value *h* at time *t* consisting of an amount $\Delta_{1}^{\left(h\right)}(t)$ of stock and an amount $\Delta_{2}^{\left(h\right)}(t)$ of the risk-free bond: $h=\Delta_{1}^{\left(h\right)}(t)s+\Delta_{2}^{\left(h\right)}(t)b$. To replicate options, suppose that at time *t* an amount equal to the value of the portfolio *h* is invested in the options. If this investment is self-financing, then, after a small increment in time *ϵ*, the value of the investment will be (*h*/*c*)*C*. The moments of *h* in terms of *c* are therefore ${\left(h/c\right)}^{n}M_{c}^{\left(n\right)}(c,t,\epsilon )$. The moments of an option, $M_{c}^{\left(n\right)}(c,t,\epsilon )=\int dC{\left(C-c\right)}^{n}p(C,t+\epsilon |c,t)$, are formally the same as the right hand side of (8a), (8b), (8c) where $\Delta_{1}^{\left(f\right)}(t)=0$ and the sign of each term in the odd moments (8a), (8b), (8c) is flipped. On the other hand, the moments of *h* as a function of those of *s* and *b* are:

$$M_{h}^{\left(1\right)}\;=\;\Delta_{1}^{\left(h\right)}M_{s}^{\left(1\right)}+\Delta_{2}^{\left(h\right)}M_{b}^{\left(1\right)},$$ (9a)

$$M_{h}^{\left(2\right)}\;=\;{\left(\Delta_{1}^{\left(h\right)}\right)}^{2}M_{s}^{\left(2\right)},$$ (9b)

$$M_{h}^{\left(3\right)}\;=\;{\left(\Delta_{1}^{\left(h\right)}\right)}^{3}M_{s}^{\left(3\right)}.$$ (9c)

In the construction of this portfolio, it will also be assumed that the KM expansion for the stock *s*(*t*) is truncated after the third term, i.e. $M_{s}^{\left(4\right)}=M_{s}^{\left(5\right)}=\ldots =0$. By choosing during each interval of time [*t*, *t* + Δ*t*), Δ*t* > 0, $\Delta_{1}^{\left(h\right)}(t)=(h/c)(\partial c/\partial s)$ and $\Delta_{2}^{\left(h\right)}(t)=\left(h-\Delta_{1}^{\left(h\right)}(t)s\right)/b$, such that the portfolio equation holds at *t*, the fluctuations of the two investments are approximately balanced. The approximation is stronger as the ratio *R*,

$$R=\frac{M_{s}^{\left(3\right)}\frac{\partial c}{\partial s}\frac{\partial^{2}c}{\partial s^{2}}}{2M_{s}^{\left(2\right)}{\left(\frac{\partial c}{\partial s}\right)}^{2}},$$ (10)

becomes smaller, *R* ≪ 1. The ratio *R* is a measure of the accuracy of replication in the ORP method (for a detailed discussion on *R*, see *Supporting Information*, S1 Fig. and S2 Fig.). The *no-arbitrage* condition requires the two investments have equal returns:

$$M_{h}^{\left(1\right)}=\frac{h}{c}M_{c}^{\left(1\right)}.$$ (11)

In the classic Black-Scholes-Merton theory, the DHP and ORP methods are perfectly equivalent yielding compatible systems of equations with the same solution. However, when third-order noise is taken into account, the ORP fails to provide a compatible system of equations. Surprisingly, the truncation from infinity down to the third term keeps the system of equations resulting from the DHP approach compatible, so, even with third-order noise, the fluctuations of an option and stock portfolio can still be canceled.

### Price of option written on underlier with skewed probability distribution

We will motivate here a particular choice for the functional form of the transition moments used above. In terms of the transition probability per unit time, *W*, the *n*th derivate transition moment for an asset *z* is $D_{z}^{\left(n\right)}=(1/n!)\int \rho^{n}W(\rho ;z)d\rho$. This leads to the derivate transition moments for the percent return $D_{z}^{\left(n\right)}/z^{n}$ = (1/*n*!) *∫* *κ* ^*n*^ *W*(*κ*; *z*)*dκ* through a change in variables from the price jump *ρ* to the percent return *κ*(*ρ*) = *ρ*/*z*. Recall that *p*(*κ*; *z*)*dκ* = *p*(*ρ*; *z*)*dρ* for the transition probabilities implies *W*(*κ*; *z*)*dκ* = *W*(*ρ*; *z*)*dρ*.

Using a *no-arbitrage* argument, the derivate transition moments for the percent return should be independent of the nominal value of the asset *z*. There are multiple ways to get market exposure to the same asset, so the risk-return characteristics should not be a function of the asset price. Then, $D_{z}^{\left(n\right)}\propto z^{n}$.

The derivate transition moments and the transition moments for the stock become thus $D_{s}^{\left(n\right)}=(1/n!)\xi_{n}s^{n}$, $M_{s}^{\left(n\right)}=\xi_{n}s^{n}\epsilon$. To use the familiar notation in the literature, let *ξ*_1_ = *μ*, *ξ*_2_ = *σ* ^2^ and the new third-order parameter *ξ*_3_ = *γ* ^3^. For bonds, $D_{b}^{\left(1\right)}=rb$, $M_{b}^{\left(1\right)}=rb\epsilon$ and $M_{b}^{\left(n\right)}$ and $D_{b}^{\left(n\right)}$ are zero for *n* ≥ 2.

The partial differential equation governing the option price *c*(*s*, *t*) is obtained using using either (7) or (11), dividing by *ϵ* and taking the limit *ϵ* → 0

$$\frac{\partial c}{\partial t}+rs\frac{\partial c}{\partial s}+\frac{1}{2}\sigma^{2}s^{2}\frac{\partial^{2}c}{\partial s^{2}}+\frac{1}{6}\gamma^{3}s^{3}\frac{\partial^{3}c}{\partial s^{3}}-rc=0.$$ (12)

The boundary conditions for an European-style call option [1, 2] are: *c*(0,*t*) = 0 for all *t*, *c*(*s*, *t*) → *s* as *s* → ∞ and *c*(*s*, *T*) = max{*s*−*K*,0}. Using the change of variables, (2), and *u*(*x*, *τ*) = *c*(*s*, *t*)exp(*rτ*), the equation (12) becomes

$$\frac{\partial u}{\partial \tau }=\frac{1}{2}(\sigma^{2}-\gamma^{3})\frac{\partial^{2}u}{\partial x^{2}}+\frac{1}{6}\gamma^{3}\frac{\partial^{3}u}{\partial x^{3}},$$ (13)

with solution

u(x,τ)=∫u(φ,0)G(x−φ,τ)dφ. (14)

Here, *u*(*ϕ*,0) = *K*(*e* ^max{*ϕ*,0}^−1) and the Green function *G* is given by

$$G(x,\tau )\;=\;\pm {\left(3\tau n\right)}^{-\frac{1}{3}}\exp\left(\frac{2m^{3}}{3^{3}n^{2}}\tau -\frac{m}{3n}x\right)\text{Ai}\left(m^{2}{\left(3n\right)}^{-\frac{4}{3}}\tau^{\frac{2}{3}}-{\left(3\tau n\right)}^{-\frac{1}{3}}x\right).$$ (15)

with ± = sign(*γ*) and Ai(*x*) is the Airy function [19]. Therefore, the solution takes the form in (1).

The solution *c*(*s*, *t*) represents a valid price only if it is a positive function. This will happen if *u*(*x*, *τ*) is positive. It turns out that *u*(*x*, *τ*) ≥ 0 for all *γ* > 0 (see *Supporting Information*). On the other hand, for *γ* < 0, the price is positive for all pairs in the upper plane (*a* ≥ 0,*τ*) but becomes negative for regions in the lower plane (*a* < 0,*τ*). However, for (*a* < 0,*τ*) the negative prices predicted by (1) are very small, so it can be assumed that the cost is virtually zero. Avoiding the region (*a* < 0,*τ*) imposes a condition on the moneyness, *M* = ln(*s*/*K*), of the option. *Moneyness* is a measure of how much “in-the-money” (*s* exceeds *K*) or “out-of-the-money” (*K* exceeds *s*) the option is. In other words, it provides a measure of how valuable the option is. Excluding the region *a* < 0 implies a “minimum allowable moneyness” condition. Namely, for *γ* < 0 the option price is guaranteed to be positive for all (*s*, *τ*) for which the moneyness *M* > *M*_min_ with

$$M_{\min}(\tau |\sigma ,\gamma ,r)=-\left(r+\frac{1}{2}\sigma^{2}-\frac{1}{6}\gamma^{3}-\frac{1}{2}\frac{\sigma^{4}}{\gamma^{3}}\right)\tau .$$ (16)

**Figure 1:**
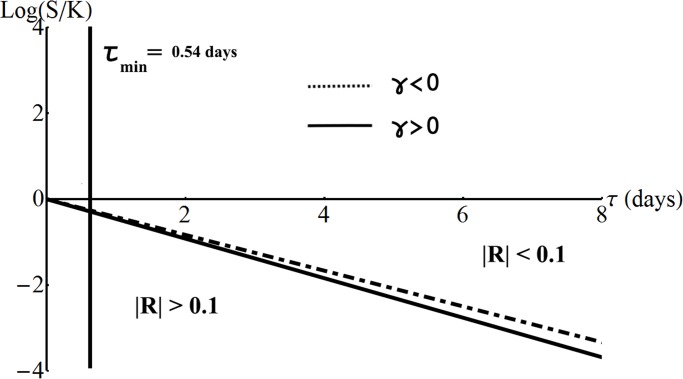
ORP replication accuracy region. Approximation for the accuracy region ∣*R*∣ < *R*_*c*_ in the (*M*, *τ*)–plane for *σ* = 0.2 years^−1/2^, *γ* = ±0.01 years^−1/3^, *r* = 0.02 years^−1^, *R*_*c*_ = 0.1 and *η*_*c*_ = 6.

As *γ* → 0^−^, the minimum threshold *M*_min_ → −∞, implying that the option price becomes positive for all (*s*, *τ*), recovering the Black-Scholes-Merton result. Another minimum moneyness condition appears from a completely different reason and only for the ORP method. The measure *R* that quantifies the accuracy of the replication, (10), should be small, ∣*R*∣ < *R*_*c*_. The constant *R*_*c*_ is a predefined level of precision accepted by the market participant. Fig. 1 helps to locate the region ∣*R*∣ < *R*_*c*_ in the plane moneyness-time to maturity, (ln(*s*/*K*), *τ*).

The region must be above the curve *R* = *R*_*c*_ which starts at *τ* = *T* at a low negative moneyness, implying that the replication is valid for stock prices covering the whole range from close to zero to infinity. As the time to maturity decreases, the curve has a linear growth.

This means that, for a given time to maturity *τ*, and for a given set of parameters (*σ*, *γ*, *r*), the replication is better than *R*_*c*_ if the stock price *s* at *τ* takes a value such that the moneyness is above a minimum threshold, ln(*s*/*K*) > *M*_min_(*τ*∣*σ*, *γ*, *r*) given by

$$M_{\min}=-\left(r+\frac{1}{2}\sigma^{2}-\frac{1}{6}\gamma^{3}\pm R_{c}\left(1\pm \frac{R_{c}}{2}\right)\frac{\sigma^{4}}{\gamma^{3}}\right)\tau ,$$ (17)

with ± = sign(*γ*).

Close to the expiration time, the curve ∣*R*∣ = *R*_*c*_ changes from a linear growth to an exponential one. This change occurs around a *τ*_min_ and the exponential growth of the curve is confined to the left of the vertical line at *τ*_min_. For *τ* < *τ*_min_ the replication is accurate only for large stock prices. Therefore, a simple approximation is to restrict the region of high ORP replication accuracy to the right of a vertical line located at a *τ*_min_. In Fig. 1, *τ*_min_ = 13 hours and was found from *τ* = 2(*γ*/*σ*)^6^ *η* ^3^ with *η* = 6 (see *Supporting Information*). For precise results, a one-time numerical tabulation of the curve *R* = *R*_*c*_ can be performed in the plane (*η*, *a*) and transformed in the plane (ln(*s*/*K*), *τ*) for given (*σ*, *γ*, *r*). An analysis of the minimum moneyness condition for various *σ* and *γ* is shown in S3 Fig. of the *Supporting Information*.

## Numerical example

The presence of third-order noise in the stochastic process used to describe the option’s underlier can lead to sizable option price differentials relative to the Black-Scholes predicted valuations. This section explores the difference in the option prices derived using the model in this manuscript and the traditional Black-Scholes approach. We consider European call options written on the publicly traded stock of General Electric Company (NYSE:GE). The choice in security was motivated by its very high trading volume (a 3-month average of roughly 27.9M [20]).

For the estimation of the 2 unobservable parameters *σ* and *γ* in (1), we used daily end-of-day closing prices [20] between 2010/8/15 and 2014/8/15. We segmented the data set consisting of approximately 1000 points in 10 bins. For each bin, the averages ⟨*ρ* ^2^⟩/⟨*z* ^2^⟩ and ⟨*ρ* ^3^⟩/⟨*z* ^3^⟩ were taken, where *ρ* is the price jump and *z* is the is the asset price before the jump. The values obtained appeared stable, which confirms the second and third derivate transition moments’ dependency on *z* ^2^ and *z* ^3^, respectively. The values for *σ* and *γ* were finally obtained by taking the simple average of the previously obtained values of each bin’s averages. The risk-free rate *r* was taken at 3.5%, the average daily yield on the 30-year U.S. Government Bond during the same time period [21].

**Figure 2:**
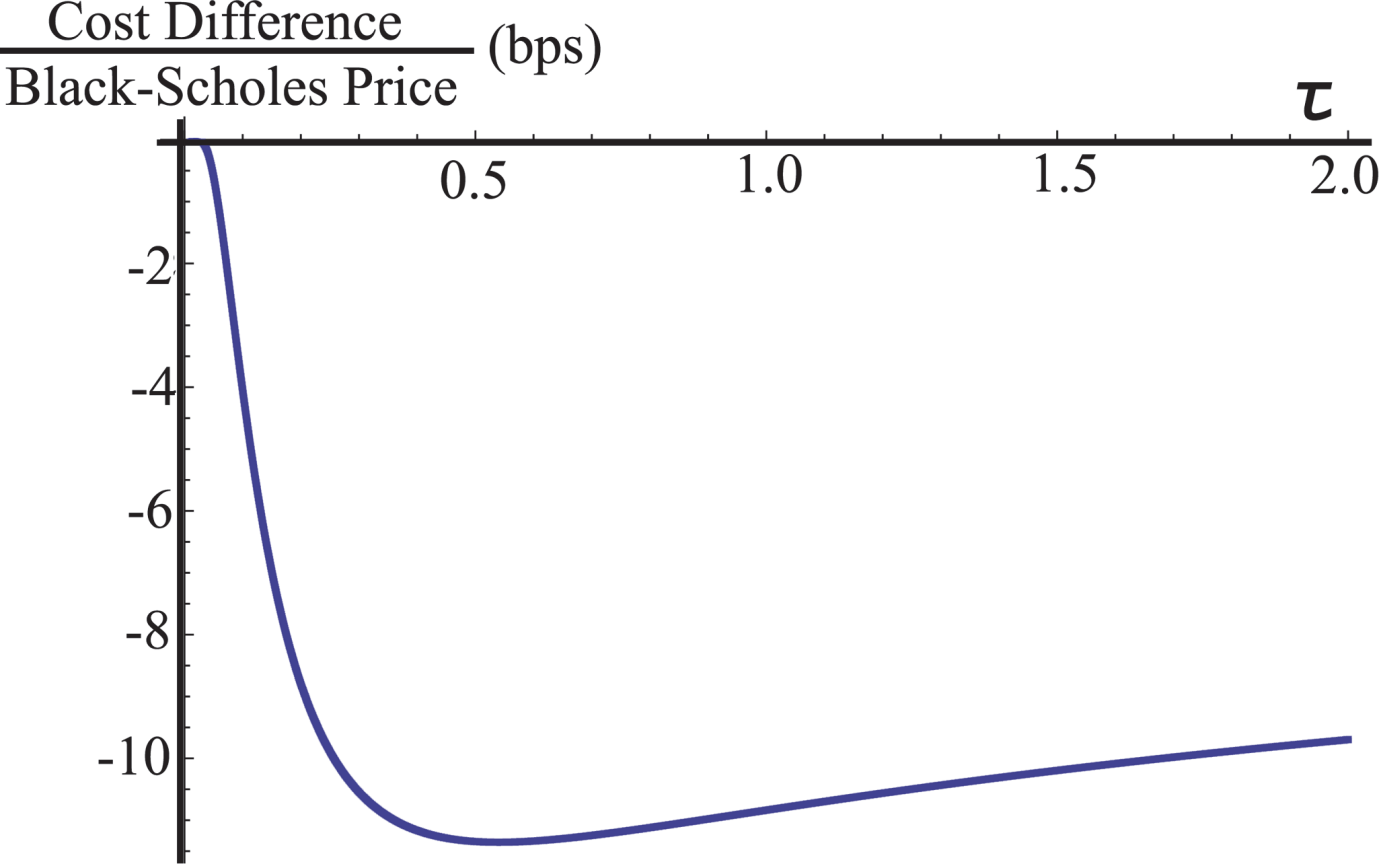
Option price difference for different maturities. Difference in option prices (proposed vs. Black-Scholes) expressed as a percent of the Black-Scholes price for different times to maturity *τ*, where *K* = 21 USD, *σ* = 0.265 years^−1/2^, *γ* = 0.068 years^−1/3^ and *r* = 0.035 years^−1^.

**Figure 3:**
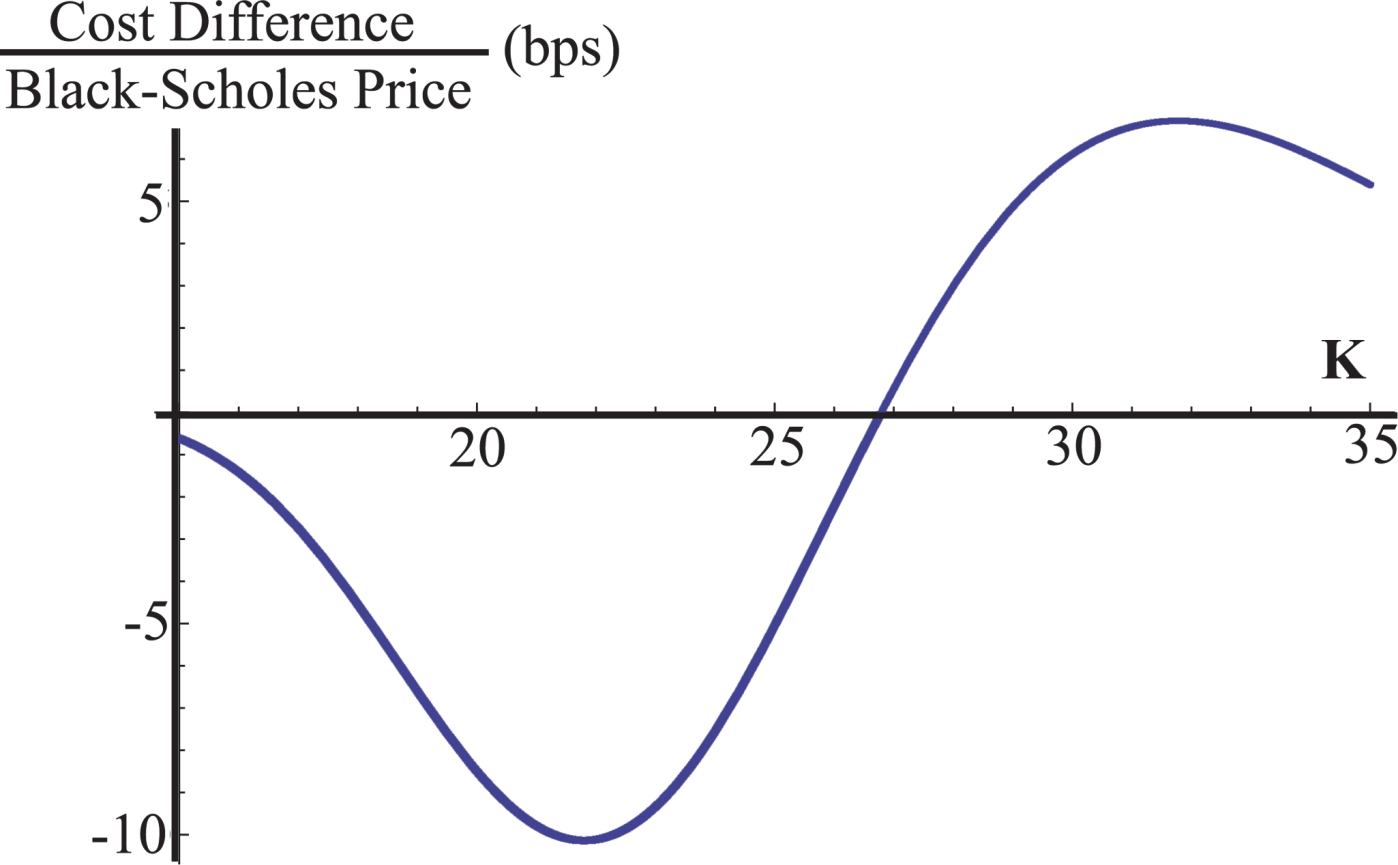
Option price difference for different strikes. Difference in option prices (proposed vs. Black-Scholes) expressed as a percent of the Black-Scholes price for different strikes *K*, where *τ* = 0.5 years, *σ* = 0.265 years^−1/2^, *γ* = 0.068 years^−1/3^ and *r* = 0.035 years^−1^.

To understand the difference (see Table 1 and Table 2) between the option prices derived using the proposed model and the Black-Scholes-Merton theory, we look at this difference expressed as a fraction of the Black-Scholes price. One can see in Fig. 2 that the price difference reaches a minimum of about −11 basis points (bps) as the time to maturity falls to about 0.5 years, given that the option is $4 in-the-money. Now, fixing the maturity of the option to 0.5 years, Fig. 3 shows the dependency of the price difference on the strike price *K*. It can be seen that, when the option is $3 in-the-money, the proposed price is 10 bps under the Black-Scholes price, but, when the option is $6 out-of-the-money, the sign flips and the proposed price is 7 bps above.

**Table 1:** Option price (USD) comparison for different maturities. Difference in option prices (proposed vs. Black-Scholes) for different times to maturity *τ*, where *K* = 21 USD, *σ* = 0.265 years^−1/2^, *γ* = 0.068 years^−1/3^ and *r* = 0.035 years^−1^.

**Option Maturity**	**Proposed Price**	**Black-Scholes Price**
0.25	4.2942	4.2984
0.50	4.6975	4.7029
0.75	5.0837	5.0894
1.00	5.4439	5.4498
1.25	5.7810	5.7871
1.50	6.0986	6.1049
1.75	6.3998	6.4062
2.00	6.6868	6.6933

**Table 2:** Option price (USD) comparison for different strikes. Difference in option prices (proposed vs. Black-Scholes) for different strikes *K*, where *τ* = 0.5 years, *σ* = 0.265 years^−1/2^, *γ* = 0.068 years^−1/3^ and *r* = 0.035 years^−1^.

**Option Strike**	**Proposed Price**	**Black-Scholes Price**
15	10.2625	10.2628
18	7.3598	7.3623
21	4.6975	4.7029
24	2.6055	2.6096
27	1.2549	1.2546
30	0.5332	0.5299
33	0.2044	0.2007
36	0.0722	0.0697

## Conclusion

A third-order KM expansion was used to describe the distribution of stock prices. This allowed for the introduction of skewness (third-order noise) in the price distribution. The price of European-style call options written on stocks governed by the truncated KM expansion was then derived using two methods. In the first, it was shown that a portfolio which is short one unit of the option and long a varying amount of the underlying stock (*i.e.*, a *delta-hedged portfolio*) can be constructed to return the risk-free return. In the second, a portfolio consisting of a varying amount of a stocks and risk-free bonds was constructed to have the same fluctuations (moments higher than the first) as a capital-equivalent investment in options. In both methods, the assumption that the option is a function of the underlier’s price and of time led to a partial differential equation for the option price. This equation can be solved in closed form. A surprising result emerged in the analysis of the two methods described above. Different than a usual second-order truncation of the KM expansion, as in the Black-Scholes-Merton theory, the two methods are no longer equivalent. The introduction of a third-order moment only approximately equates the risk of the stock-bond portfolio to that of the option. This approximation was explored analytically and a measure for the replication accuracy *R* was developed. However, the fluctuations of the option-stock portfolio do cancel out even with the third order included.

## Supporting Information

S1 Supporting Information Supporting Information. (PDF)

S1 Fig The case *γ* > 0. The curve *R*(*η*, *a*) = *R*_*c*_ = 0.1, connected line, is approximated by two curves, namely the crossed line for *a* < 0 and the parabola, dashed line, for *a* > 0. The shaded region corresponds to *R* < 0.1.
(TIF)

S2 Fig The case *γ* < 0. The curve *R*(*η*, *a*) = *R*_*c*_ = 0.1, connected line, is approximated by the parabola, dashed line, for *a* > 0. The region *a* < 0 is not used because the cost can take negative values, although close to zero. The shaded region corresponds to *R* < 0.1.
(TIF)

S3 Fig Area where *η* > *η*_*c*_. The region above the curves shows combinations of (*σ*, *γ*) for which *η* > *η*_*c*_ and *M*_min_ < *M*_*c*_ for *τ* = 14 days, *R*_*c*_ = 0.1, *η*_*c*_ = 10, *M*_*c*_ = 1/100 and *r* = 0.02 years^−1^.
(TIF)
