# Supplementary material for: A Kramers-Moyal Approach to the Analysis of Third-Order Noise with Applications in Option Valuation
Source: PLoS One. 2015 Jan 27;10(1):e0116752. doi: 10.1371/journal.pone.0116752 (PMC4308111; doi:10.1371/journal.pone.0116752)
Supplement: S1 Supporting Information — (PDF) [file pone.0116752.s001.pdf]

Supporting Information for "A Kramers-Moyal approach to the analysis of third-order noise with applications in option valuation"

Dan M. Popescu[1], Ovidiu Lipan[1*]
1 Department of Physics, University of Richmond, Richmond, Virginia, United States of America
$\ast$ E-mail: olipan@richmond.edu

Rationale for the explicit forms of the derivate transition moments

The functional form of the derivate transition moments is justified in this section. The derivate moments for the instantaneous return $\kappa = z(t+\epsilon)/z(t) - 1$ on an asset z are given by

$$\frac{D^{(n)}(z)}{z^n} = \frac{1}{n!}\int_{-\infty}^{\infty} \kappa^n W(\kappa; z) d\kappa, \tag{1}$$

where $W(\kappa; z)$ is the *transition probability per unit time* [1].

To illustrate that the RHS should not depend on the nominal value of z, consider the example of an investor who wants to buy an index (*e.g.*, S&P 500 – Standard and Poor's 500) and can get market exposure either through Exchange Traded Funds — for example, the SPDR S&P 500 ETF Trust (VOO) traded on the New York Stock Exchange — or through mutual funds — *e.g.*, the Vanguard 500 Index Funds (VFINX). Although the nominal value of a share in the ETF is considerably different than that of a share in the mutual fund, the market exposure to be the same. That is, the two different investments should have the same risk-return characteristics, independent of the value z. Hence, the derivate moments of the returns will not depend on the asset price. Mathematically, $D^{(n)}(z) \propto z^n$.

Discussion on the positivity of the solution

In this section, proofs are provided for the positivity of $c(s,t)$ for the case $\gamma > 0$ and regions of $\gamma < 0$.

The case $\gamma > 0$ implies β, $n > 0$, and therefore for $x \leq 0$, the integral

$$u(x,\tau) = \int_{-\infty}^{\infty} u(\phi, 0) G(x-\phi, \tau) d\phi, \tag{2}$$

is performed over a product of positive functions, so the cost $u(x,\tau)$ is positive.

For the case $x > 0$, it will be shown that $\frac{\partial u}{\partial x} \geq 0$ and together with $u(0,\tau) \geq 0$ (inferred from the conclusion above), it follows that $u(x,\tau) \geq 0$. During the analysis, we distinguish between $a > 0$ and $a < 0$.

The derivative of the price is

$$\frac{\partial u}{\partial x} = K e^{-\frac{1}{3}(\eta-\beta)^3} e^{-\beta a} \int_a^{\infty} e^{\eta\zeta} \mathrm{Ai}(\zeta) d\zeta. \tag{3}$$

Therefore, the sign of $\frac{\partial u}{\partial x}$ will be given by the sign of the integral. If $a \geq 0$,the integral is performed over a product of positive functions, since $\mathrm{Ai}(\zeta) > 0$ for $\zeta \geq 0$.

Now consider the case $a < 0$. Split up the integral in the following way

$$\int_a^{\infty} e^{\eta\zeta} \mathrm{Ai}(\zeta) d\zeta = \int_a^{0} e^{\eta\zeta} \mathrm{Ai}(\zeta) d\zeta + \int_0^{\infty} e^{\eta\zeta} \mathrm{Ai}(\zeta) d\zeta. \tag{4}$$

The second integral in (4) is again taken over positive functions, so it is positive. Before gauging the sign of the first integral, we note that $\eta \geq 0$. Integrating by parts,

$$\int_a^0 e^{\eta\zeta}\mathrm{Ai}(\zeta)d\zeta = \int_a^0 e^{\eta\zeta}\mathrm{Aip}'(\zeta)d\zeta = -\mathrm{Aip}(a)e^{\eta a} + \eta\int_a^0 \left(-\mathrm{Aip}(\zeta)\right)e^{\eta\zeta}d\zeta. \tag{5}$$

Here $\mathrm{Aip}(x)$ is the primitive of the Airy function

$$\mathrm{Aip}(x) = \int_0^x \mathrm{Ai}(\xi)d\xi, \tag{6}$$

which is known to be positive for positive x and negative for negative x. The first term in (5) is positive, since $-\mathrm{Aip}(a) > 0$ for $a < 0$. The second term contains an integral taken over a product of positive functions and a positive parameter η.

For the case $\gamma < 0$, it is more convenient to view the solution u as a function of a and τ:

$$u(a,\tau) = Ke^{-\frac{1}{3}(\eta-\beta)^3}\int_{-\infty}^a d\chi\left(e^{-\beta a+\eta\chi} - e^{(-\beta+\eta)\chi}\right)\mathrm{Ai}(\chi). \tag{7}$$

We distinguish between $a > 0$ and $a < 0$. For $a > 0$, we will show that $\frac{\partial u}{\partial a} \geq 0$ and $u(0,\tau) \geq 0$ to conclude that $u(0,\tau) \geq 0$. For $a < 0$, we will show that $u(a,\tau)$ is small and so the cost of the option is close to zero. First, we prove that u is positive for $a = 0$. We have

$$u(0,\tau) = Ke^{-\frac{1}{3}(\eta-\beta)^3}\left(\int_{-\infty}^0 d\chi e^{\eta\chi}\mathrm{Ai}(\chi) - \int_{-\infty}^0 d\chi e^{(-\beta+\eta)\chi}\mathrm{Ai}(\chi)\right). \tag{8}$$

We will use the Laplace transform of the Airy function (see [2])

$$\int_0^\infty e^{-pt}\mathrm{Ai}(-t)dt = \frac{1}{3}e^{\frac{p^3}{3}}\left(\frac{\Gamma\left(\frac{1}{3},\frac{p^3}{3}\right)}{\Gamma\left(\frac{1}{3}\right)} + \frac{\Gamma\left(\frac{2}{3},\frac{p^3}{3}\right)}{\Gamma\left(\frac{2}{3}\right)}\right), \tag{9}$$

for $\Re p > 0$, where $\Gamma(s)$ is the Gamma function and $\Gamma(s,x)$ is the incomplete Gamma function $\Gamma(s,x) = \int_x^\infty t^{s-1}e^{-t}dt$. Using the Laplace transform, (8) becomes

$$u(0,\tau) = \frac{1}{3}Ke^{-\frac{1}{3}(\eta-\beta)^3} \left(\frac{e^{\frac{\eta^3}{3}}\Gamma\left(\frac{1}{3},\frac{\eta^3}{3}\right) - e^{\frac{(\eta-\beta)^3}{3}}\Gamma\left(\frac{1}{3},\frac{(\eta-\beta)^3}{3}\right)}{\Gamma\left(\frac{1}{3}\right)} + \frac{e^{\frac{\eta^3}{3}}\Gamma\left(\frac{2}{3},\frac{\eta^3}{3}\right) - e^{\frac{(\eta-\beta)^3}{3}}\Gamma\left(\frac{2}{3},\frac{(\eta-\beta)^3}{3}\right)}{\Gamma\left(\frac{2}{3}\right)}\right). \tag{10}$$

Now, consider the function

$$h(x) = e^x\Gamma(s,x) \text{ for } x > 0 \text{ and } 0 < s < 1. \tag{11}$$

The function $h(x)$ is decreasing because

$$h'(x) = e^x\int_x^\infty \left(t^{s-1}e^{-t} - x^{s-1}e^{-x}\right)dt < 0. \tag{12}$$

Therefore, given that $\eta \geq 0$ and $\beta \leq 0$, the two numerators of the fractions enclosed in brackets in (10) are positive and, hence, $u(0,\tau) \geq 0$.

We can now show that u is in fact positive for all $a \geq 0$. Use (7) to find

$$\frac{\partial u}{\partial a} = K e^{-\frac{1}{3}(\eta-\beta)^3}(-\beta)e^{-\beta a}\left(\int_{-\infty}^{0} d\chi e^{\eta\chi}\mathrm{Ai}(\chi) + \int_{0}^{a} d\chi e^{\eta\chi}\mathrm{Ai}(\chi)\right). \tag{13}$$

The first integral is the Laplace transform of the Airy function given by (9), which is positive. The second integral is also positive, because it is performed over a product of positive functions (the Airy function is positive for positive argument). Therefore, u is increasing in a and, combining this result with $u(0,\tau) \geq 0$, we get that $u(a,\tau) \geq 0$ for $a \geq 0$.

In the case $a < 0$, there are regions where the cost $u(a,\tau)$ becomes negative. However, the cost for $a < 0$ takes only on small values because the integral in (7) decreases as $\exp\left((-\beta+\eta)a\right)$ multiplied with $\exp\left(-1/3(-\beta+\eta)^3\right)$. The point $a = 0$ corresponds to a certain lower bound for the price of the underlier s. If s goes below this threshold, the third-order approximation fails to give a positive cost if $\gamma < 0$. Because the cost is small for $a < 0$, we will assume $c(s,t) = 0$ for this case.

The limiting case $\gamma \to 0$

The asymptotic form of the option pricing formula for a small skewness parameter γ is derived in this section. In the limit $\gamma \to 0$, we recover the classic Black-Scholes-Merton cost. The change of variables

$$y = \frac{x}{\sqrt{\frac{1}{2}(\sigma^2 - \gamma^3)}}, \tag{14}$$

transforms the equation for the option cost into the form

$$\frac{\partial u(y,\tau)}{\partial \tau} = \frac{\partial^2 u}{\partial y^2} + c\frac{\partial^3 u}{\partial y^3}, \tag{15}$$

where

$$c = \frac{1}{6}\left(\frac{\gamma}{\sqrt{\frac{1}{2}\left(\sigma^2 - \gamma^3\right)}}\right)^3. \tag{16}$$

As $\gamma \to 0$, so does c, and as $c \to 0$, (16) goes into the heat equation, which is the Black-Scholes-Merton equation after a change of variables. The corrections to Black-Scholes-Merton equation will therefore be expressed in terms of c. Start with the cost is expressed as

$$u(y,\tau) = \int_{0}^{\infty} d\zeta u(\zeta,0) G(y-\zeta,\tau), \tag{17}$$

with $u(\zeta,0) = K\left(e^{\max\{\zeta,0\}} - 1\right)$. The Green function in the variable y and for $\gamma > 0$ is given by:

$$G(y,\tau) = (3\tau c)^{-1/3}\exp\left(\frac{2}{3^3c^2}\tau - \frac{1}{3c}y\right)\mathrm{Ai}\left((3c)^{-4/3}\tau^{2/3} - (3\tau c)^{-1/3}y\right). \tag{18}$$

The Airy function has two different asymptotic expansions corresponding to its argument going to ∞ or $-\infty$. Hence, to get the asymptotic value for the cost, we need to find the regions for which the argument of the Airy function is positive or negative. Let the argument of the Airy function be

$$(3c)^{-\frac{4}{3}}\tau^{\frac{2}{3}} - (3\tau c)^{-\frac{1}{3}}(y-\zeta) = (3c)^{-\frac{4}{3}}\left(\tau^{\frac{2}{3}} - \frac{3(y-\zeta)}{\tau^{\frac{1}{3}}}c\right) \equiv \theta. \tag{19}$$

For $\gamma > 0$, the argument tends to $+\infty$ for any ζ. Indeed, for a fixed τ and y, if $y < 0$, the argument is positive for any $\zeta > 0$ because $c > 0$. For $y > 0$, it is sufficient to take $c < 3y/\tau$ to get a positive argument for any $\zeta > 0$. We should, therefore, use the asymptotic expression [2] of $\mathrm{Ai}(\theta)$ for $\theta \to +\infty$

$$\mathrm{Ai}(\theta) = \frac{1}{2\pi} e^{-\frac{2}{3}\theta^{3/2}} \theta^{-1/4} \int_{-\infty}^{\infty} e^{-v^2} \cos\left(\frac{v^3}{3\theta^{3/4}} dv\right), \tag{20}$$

in the Green function, (20)

$$G(y,\tau) = \frac{1}{2\pi}\tau^{-1/3} \exp\left(\frac{2}{3^3c^2}\tau - \frac{1}{3c}y - \frac{2}{3^3c^2}\theta^{-3/2}\right)\theta^{-1/4} \int_{-\infty}^{\infty} e^{-v^2} \cos\left(\frac{v^3}{\theta^{3/4}c}\right) dv. \tag{21}$$

From a Taylor expansion of $\theta^{-3/2}$, $\theta^{-1/4}$ and the cosine around $c = 0$ and ignoring all the terms $\mathcal{O}(c^2)$, the Green function becomes

$$G(y,\tau) = \frac{1}{2\sqrt{\pi\tau}} e^{-\frac{y^2}{4\tau}} \left(1 + c\left(\frac{3y}{4\tau} - \frac{y^3}{\tau^2}\right)\right). \tag{22}$$

Using (22) in the solution (17) and changing back the variables to (c, s, t), we finally get

$$\begin{aligned} c(s,t) = s\frac{1}{2\pi}\int_{-\infty}^{d_+} d\theta \left(1 + \gamma^3\Omega\left(\theta - \sqrt{(\sigma^2-\gamma^3)\tau}\right)\right) e^{-\theta^2/2} - \\ Ke^{-r(T-t)}\frac{1}{2\pi}\int_{-\infty}^{d_-} d\theta \left(1 + \gamma^3\Omega(\theta)\right) e^{-\theta^2/2}, \end{aligned} \tag{23}$$

where

$$d_\pm = \frac{\ln\left(\frac{s}{K}\right) + \left(r \pm \frac{1}{2}\left(\sigma^2 - \gamma^3\right)\right)\tau}{\sqrt{(\sigma^2-\gamma^3)\tau}}, \tag{24}$$

and the correction term

$$\Omega(\theta) = \frac{1}{4}\frac{\theta}{\sqrt{(\sigma^2-\gamma^3)\tau}}\left(1 - \frac{\theta^2}{3}\right). \tag{25}$$

We can now see that as $\gamma^3 \to 0$, the $d_\pm$ become the ones in the Black-Scholes-Merton model and the correction term $\gamma^3\Omega$ vanishes. The limiting result is the classic Black-Scholes-Merton formula.

For $\gamma < 0$, the argument z is positive if $\zeta < y - \frac{\tau}{3c}$ and negative for any other ζ. Consequently, we will split up the solution in (17) into two integrals

$$\begin{aligned} u(y,\tau) = \int_0^{y-\frac{\tau}{3c}} d\zeta \left(e^{\zeta\sqrt{\frac{1}{2}(\sigma^2-\gamma^3)}} - 1\right) G(y-\zeta,\tau) + \\ \int_{y-\frac{\tau}{3c}}^{\infty} d\zeta \left(e^{\zeta\sqrt{\frac{1}{2}(\sigma^2-\gamma^3)}} - 1\right) G(y-\zeta,\tau), \end{aligned} \tag{26}$$

where the Green function in variable y and for $\gamma < 0$ is

$$G(y,\tau) = (-3\tau c)^{-1/3} \exp\left(\frac{2}{3^3c^2}\tau - \frac{1}{3c}y\right) \mathrm{Ai}\left((3c)^{-4/3}\tau^{2/3} - (3\tau c)^{-1/3}y\right). \tag{27}$$

Using (20) for the first integral and applying the same method as in the case $\gamma > 0$, we get

$$G(y,\tau) = \frac{1}{2\sqrt{\pi\tau}} e^{-\frac{y^2}{4\tau}} \left(1 + c\left(\frac{3y}{4\tau} - \frac{y^3}{\tau^2}\right)\right), \tag{28}$$

Using only the first integral in (26), the same asymptotic form of the cost as for the case $\gamma > 0$ results. It is left to show that the second integral goes to 0 as $c \to 0$ and, hence, is negligible for the asymptotic form of the cost. Let

$$I \equiv \int_{y-\frac{\tau}{3c}}^{\infty} d\zeta \left(e^{\zeta\sqrt{\frac{1}{2}(\sigma^2-\gamma^3)}} - 1\right) G(y-\zeta,\tau). \tag{29}$$

Since $c \to 0$ from the left, for any y and τ we can find a c so that the integral is performed over positive ζ. Let $m = 2^{-1}(\sigma^2 - \gamma^3)$ and write

$$\begin{aligned}
|I| &= \int_{y-\frac{\tau}{3c}}^{\infty} d\zeta \left(e^{\zeta\sqrt{m}} - 1\right) |G(y-\zeta,\tau)| \\
&= \int_{y-\frac{\tau}{3c}}^{\infty} d\zeta \left(e^{\zeta\sqrt{m}} - 1\right) (3\tau c)^{-1/3} \exp\left(\frac{2}{3^3c^2}\tau - \frac{1}{3c}(y-\zeta)\right) \\
&\quad \left|\mathrm{Ai}\left((3c)^{-4/3}\tau^{2/3} - (3\tau c)^{-1/3}(y-\zeta)\right)\right| \\
&\le \int_{y-\frac{\tau}{3c}}^{\infty} d\zeta \left(e^{\zeta\sqrt{m}} - 1\right) (3\tau c)^{-1/3} \exp\left(\frac{2}{3^3c^2}\tau - \frac{1}{3c}(y-\zeta)\right) \\
&= 3^{2/3}\tau^{-2/3}e^{-\frac{\tau}{3^3c^2}} \left(1 - \frac{\exp\left(\sqrt{m}\left(y-\frac{\tau}{3c}\right)\right)}{1+3\sqrt{m}c}\right).
\end{aligned}$$

From the last expression, we get

$$|I| \sim c^{\frac{2}{3}} e^{\left(-\frac{1}{c^2}-\frac{1}{c}\right)} \to 0 \text{ as } c \to 0. \tag{30}$$

Therefore, the classical Black-Scholes-Merton model is recovered as well for the case $\gamma < 0$.

The measure of accuracy R in option replication

This section provides additional information regarding the ORP replication accuracy R. It is more transparent to work in the (η, a) plane and subsequently transform the results into the moneyness-maturity plane. To this end, the option pricing formula brings R to the form

$$R(\eta,a) = \frac{e^{\eta a}\mathrm{Ai}(a)}{\eta\int_a^{\infty} e^{\eta\xi}\mathrm{Ai}(\xi)d\xi}, \text{ for } \gamma > 0, \tag{31}$$

$$R(\eta,a) = \frac{e^{\eta a}\mathrm{Ai}(a)}{\eta\int_{-\infty}^{a} e^{\eta\xi}\mathrm{Ai}(\xi)d\xi}, \text{ for } \gamma < 0. \tag{32}$$

The result for R shows that all five parameters S, τ, μ, σ and γ group together in the two parameters (η, a) such that R can be considered a two-dimensional function. The boundary of the region in the plane (η, a) is defined by the implicit equation $|R(\eta, a)| = R_c$. The boundary is a curve which can be expressed either as a function of η, $a(\eta; R_c)$, or as a function of a, $\eta(a; R_c)$, both for a given R_c. Although these curves can be numerically computed, to get insight into these boundaries, it is desirable to have analytical approximations. It turns out that these approximations are parabolas or straight lines and thus easy to use. Fig. S1 for $\gamma > 0$, and Fig. S2, respectively, for $\gamma < 0$, show the curve $R(\eta, a) = 0.1$ together with an approximation to it. For both cases, in the upper-plane $a > 0$, the approximation parabola was computed starting from the upper-right region, of large a and η. The lower-plane $a < 0$ is important only for $\gamma > 0$, because, for $\gamma < 0$, the option price is taken to be zero. The approximation curve for $a < 0$, $\gamma > 0$ is found through a sequence of inequalities and is reduced in the end to a vertical boundary line.

The measure of replication accuracy for $\gamma > 0$

In what follows, we will find the approximation for the boundary curve in the upper plane $a > 0$ and, separately, in the lower plane $a < 0$. For $a \geq 0$ we have $R(\eta, a) \geq 0$ and the boundary is $R(\eta, a) = R_c$. On this boundary, $dR = 0$. For the case $\gamma > 0$,

$$\frac{\partial R}{\partial \eta} = \left(a - \frac{1}{\eta} - \eta^2\right) R - \eta \left(\eta - \frac{\mathrm{Ai}'(a)}{\mathrm{Ai}(a)}\right) R^2, \tag{33}$$

and

$$\frac{\partial R}{\partial a} = \left(\eta + \frac{\mathrm{Ai}'(a)}{\mathrm{Ai}(a)}\right) R + \eta R^2, \tag{34}$$

where integration by parts was used on the indefinite integrals. For large η and $a > 0$,

$$\frac{\mathrm{Ai}'(a)}{\mathrm{Ai}(a)} \approx -\sqrt{a} \text{ and } \frac{1}{\eta} \ll a, \tag{35}$$

so $\frac{1}{\eta}$ can be ignored, leading to

$$dR = \left(\eta + \sqrt{a}\right)\left(\left(\sqrt{a} - \eta\right) R - \eta R^2\right) d\eta - \left(\left(\sqrt{a} - \eta\right) R - \eta R^2\right) da. \tag{36}$$

From $dR = 0$ and, given that η is positive,

$$a(\eta; R_c) = \left(1 + R_c\right)^2 \eta^2. \tag{37}$$

For the case $\gamma > 0$ and in the upper plane $a > 0$ the region $R(a, \eta) < R_c$ is below the parabola in (37), Fig. S1.

If a better approximation is needed, then the region below the parabola is extended only up to the vertical line $\eta = \eta_c$ where η_c is numerically computed so that the distance between the parabola and the boundary curve $R(\eta, a) = R_c$ is less than a user predefined error.

We will argue below that, for $a < 0$, the region $|R(a, \eta)| < R_c$ is located on the right of the curve $\eta(a; R_c)$ given by $0.81\frac{1}{\eta}e^{-\frac{\eta^3}{3}}e^{-\eta|a|} = R_c$. As $a \to -\infty$, this curve decreases monotonically. It starts at $\eta(a = 0; R_c = 0.1) = 1.68$ for $R_c = 0.1$, and at 2.21 for $R_c = 0.01$. In practice, R_c is above 10 which makes $\eta(0; R_c)$ so close to zero, that the entire curve will be approximated with the vertical line segment $\eta = 0, a < 0$. If this approximation is not acceptable, then the region in the lower plane $a < 0$ should be on the right of the vertical line segment $\eta = \eta(a = 0; R_c)$. Combining with $\eta > \eta_c$, we get $\eta > \max\{\eta_c, \eta(a = 0; R_c)\}$ for the region on the right of the vertical line segment. The value $\eta_c = 6$ was used for Fig.1 of the main text.

To complete the discussion for the case $\gamma > 0$, the computations that lead to the curve $\eta(a; R_c)$ for $a < 0$ are shown. Using the result proved above:

$$\int_a^0 e^{\eta\xi}\mathrm{Ai}(\xi)d\xi \geq 0 \text{ for } a < 0, \tag{38}$$

we have

$$\left|\frac{e^{\eta a}\mathrm{Ai}(a)}{\eta\int_a^\infty e^{\eta\xi}\mathrm{Ai}(\xi)d\xi}\right| \leq \left|\frac{e^{\eta a}\mathrm{Ai}(a)}{\eta\int_0^\infty e^{\eta\xi}\mathrm{Ai}(\xi)d\xi}\right| = \frac{|e^{\eta a}\mathrm{Ai}(a)|}{\eta e^{\frac{\eta^3}{3}}\left(1 - \frac{1}{3}\left(\frac{\Gamma\left(\frac{1}{3}, \frac{\eta^3}{3}\right)}{\Gamma\left(\frac{1}{3}\right)} + \frac{\Gamma\left(\frac{2}{3}, \frac{\eta^3}{3}\right)}{\Gamma\left(\frac{2}{3}\right)}\right)\right)}$$

$$\leq \frac{3}{2}\frac{1}{\eta}e^{-\frac{\eta^3}{3}}e^{-\eta|a|}\,|\mathrm{Ai}(a)| \leq 0.81\frac{1}{\eta}e^{-\frac{\eta^3}{3}}e^{-\eta|a|}. \tag{39}$$

In terms of the time to maturity τ and the moneyness $M = \ln(s/K)$ of the option, the boundary represented by the parabola in the upper plane $(\eta, a > 0)$ and the vertical line segment $\eta = \max\{\eta_c, \eta(a=0; R_c)\}$ in the lower plane $(\eta, a < 0)$ are translated using the change in coordinates:

$$\ln(s/K) = \left(\frac{\gamma}{\sigma}\right)^6 \left(\left(-2r + \frac{1}{3}\gamma^3 - \sigma^2 + \frac{\sigma^4}{\gamma^3}\right)\eta^3 - \frac{\sigma^4}{\gamma^3} a\eta\right), \tag{40a}$$

$$\tau = 2\left(\frac{\gamma}{\sigma}\right)^6 \eta^3. \tag{40b}$$

For fixed r, σ and γ, the region below the parabola transforms into the region above the straight line shown in Fig.1 of the main text

$$M_{\min}(\tau|\sigma,\gamma,r) = -\left(r + \frac{1}{2}\sigma^2 - \frac{1}{6}\gamma^3 + R_c\left(1 + \frac{1}{2}R_c\right)\frac{\sigma^4}{\gamma^3}\right)\tau. \tag{41}$$

and the region on the right of the vertical line segment $\eta = \max\{\eta_c, \eta(a=0; R_c)\}$ transforms into the region on the right of a minimum time to maturity, $\tau > \tau_{\min}$, with $\tau_{\min} = (\gamma/\sigma)^6 \left(\max\{\eta_c, \eta(a=0; R_c)\}\right)^3$.

The measure of replication accuracy for $\gamma < 0$

The case $\gamma < 0$ is similar to $\gamma > 0$, with the exception that we need not consider the lower plane $a < 0$. Similarly to the first case, we begin by searching for the function $a(\eta; R_c)$ such that $R(\eta, a)$ from (32) is constant, $R(\eta, a) = R_c$.

Through a similar method to the one used above, we find that

$$\frac{\partial R}{\partial \eta} = \left(a - \frac{1}{\eta} - \eta^2\right) R + \eta\left(\eta - \frac{\mathrm{Ai}'(a)}{\mathrm{Ai}(a)}\right) R^2, \tag{42}$$

and

$$\frac{\partial R}{\partial a} = \left(\eta + \frac{\mathrm{Ai}'(a)}{\mathrm{Ai}(a)}\right) R - \eta R^2. \tag{43}$$

On the curve $R(a, \eta) = R_c$, using the same assumptions as in the case $\gamma > 0$,

$$a(\eta; R_c) = (1 - R_c)^2 \eta^2. \tag{44}$$

Unlike the case $\gamma > 0$, the region $R \leq R_c$ for $\gamma < 0$ is above the parabola in (44), Fig. S2.

The distance between the curve $R(a, \eta) = R_c$ and the parabola in (44) decreases as η increases. The distance will be less than a predefined error for all $\eta > \eta_c$, where η_c depends on the predefined error. In the plane $(\tau, \ln(S/K))$ the region above the parabola transforms into the region above the line

$$M_{\min}(\tau|\sigma,\gamma,r) = -\left(r + \frac{1}{2}\sigma^2 - \frac{1}{6}\gamma^3 - R_c\left(1 - \frac{1}{2}R_c\right)\frac{\sigma^4}{\gamma^3}\right)\tau, \tag{45}$$

which has the same minimum moneyness interpretation as for the case $\gamma > 0$. A better approximation will require $\tau > \tau_{\min}$ where $\tau_{\min}$ is numerically computed so that the distance between the parabola and the boundary curve $R(\eta, a) = R_c$ is less than a user predefined error, a similar approach as in the case $\gamma > 0$.

It can be seen that, for $\gamma < 0$, the minimum moneyness condition required to have a positive cost everywhere is equivalent to the minimum moneyness condition with a $R_c = 1$ to equate the fluctuations of the options to those of a stock-bond portfolio. Therefore, the positivity condition is even less restrictive than the cases analyzed in this section.

Although for $\gamma < 0$ the cost was set to zero in the lower half plane $a < 0$, it is interesting to notice that in this lower plane there are curves $a(\eta)$ for which $|R| \to \infty$. This can be understood by observing that

$$\int_{-\infty}^{a} e^{\eta\xi} \mathrm{Ai}(\xi) d\xi = 0, \tag{46}$$

which implies

$$\frac{da}{d\eta} = \eta - \frac{\mathrm{Ai}'(a)}{\mathrm{Ai}(a)}. \tag{47}$$

Inserting $\eta = 0$ in (46) we find that the curves $a(\eta)$ that fulfill (47) start on the vertical line $\eta = 0$ at the points a given by

$$\mathrm{Aip}(a) = \int_{-\infty}^{a} \mathrm{Ai}(\xi) d\xi = 0. \tag{48}$$

This shows that there is an infinite number of curves $a(\eta)$ for which the integral in the denominator of R becomes 0 and, therefore, $|R| \to \infty$.

As an additional analysis of the minimum moneyness condition, the region on the left of the curves in Fig. S3 shows the combinations of σ and γ for which $M_{\min}(\tau|\sigma, \gamma, r) < M_c$ at a given τ and for a predefined threshold M_c. In this analysis, r was considered a fixed parameter.

References

1. Gardiner CW (2004) Handbook of Stochastic Methods: for Physics, Chemistry and the Natural Sciences. Springer Series in Synergetics, Berlin, 3rd edition.

2. Vallée O, Soares M (2004) Airy Functions and Applications to Physics. Imperial College Press.

Figure legends

Figure S1. The case $\gamma > 0$. The curve $R(\eta, a) = R_c = 0.1$, connected line, is approximated by two curves, namely the crossed line for $a < 0$ and the parabola, dashed line, for $a > 0$. The shaded region corresponds to $R < 0.1$.

Figure S2. The case $\gamma < 0$. The curve $R(\eta, a) = R_c = 0.1$, connected line, is approximated by the parabola, dashed line, for $a > 0$. The region $a < 0$ is not used because the cost can take negative values, although close to zero. The shaded region corresponds to $R < 0.1$.

Figure S3. Area where $\eta > \eta_c$. The region above the curves shows combinations of (σ, γ) for which $\eta > \eta_c$ and $M_{\min} < M_c$ for $\tau = 14$ days, $R_c = 0.1$, $\eta_c = 10$, $M_c = 1/100$ and $r = 0.02\,\text{years}^{-1}$.
