# Supplementary figures and images for: A Kramers-Moyal Approach to the Analysis of Third-Order Noise with Applications in Option Valuation
Source: PLoS One. 2015 Jan 27;10(1):e0116752. doi: 10.1371/journal.pone.0116752 (PMC4308111; doi:10.1371/journal.pone.0116752)

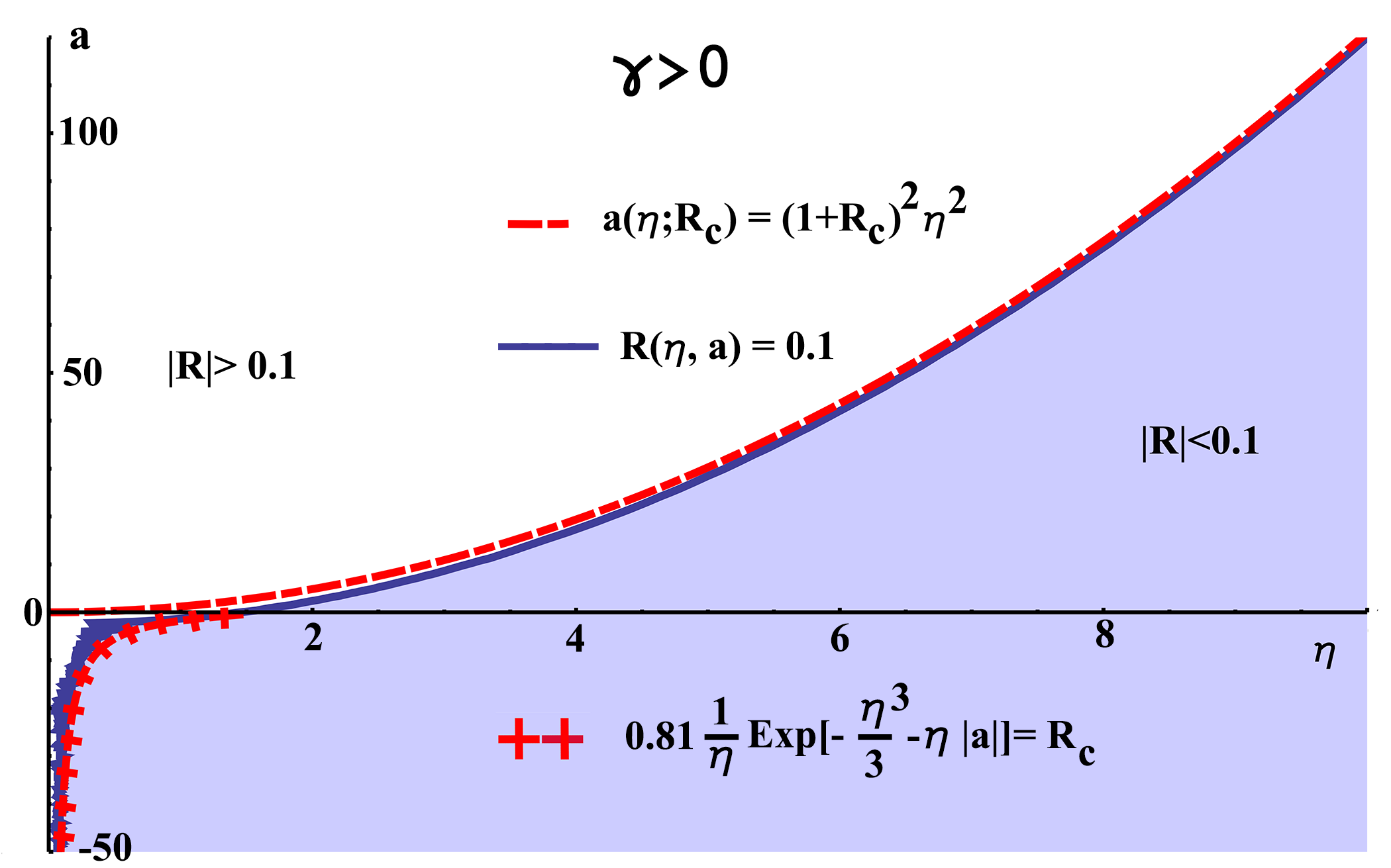

Supplement: S1 Fig — The curve R(η, a) = R c = 0.1, connected line, is approximated by two curves, namely the crossed line for a < 0 and the parabola, dashed line, for a > 0. The shaded region corresponds to R < 0.1. (TIF) [file pone.0116752.s002.tif]

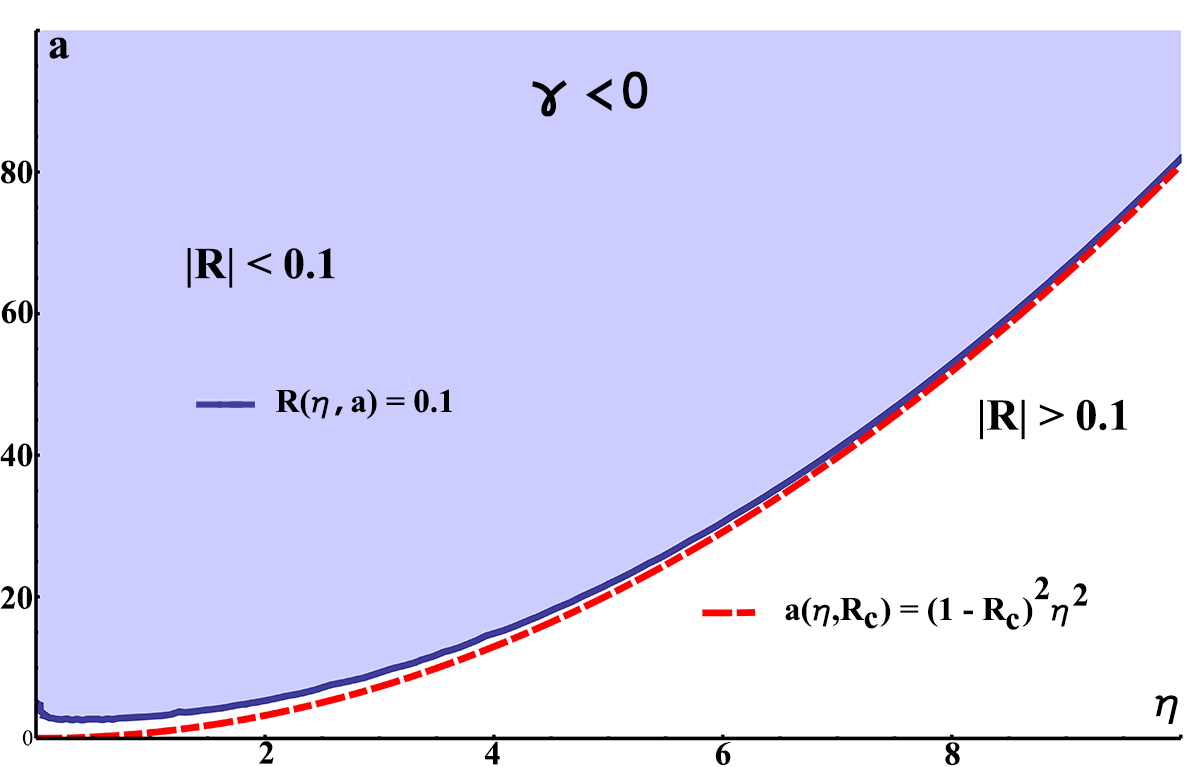

Supplement: S2 Fig — The curve R(η, a) = R c = 0.1, connected line, is approximated by the parabola, dashed line, for a > 0. The region a < 0 is not used because the cost can take negative values, although close to zero. The shaded region corresponds to R < 0.1. (TIF) [file pone.0116752.s003.tif]

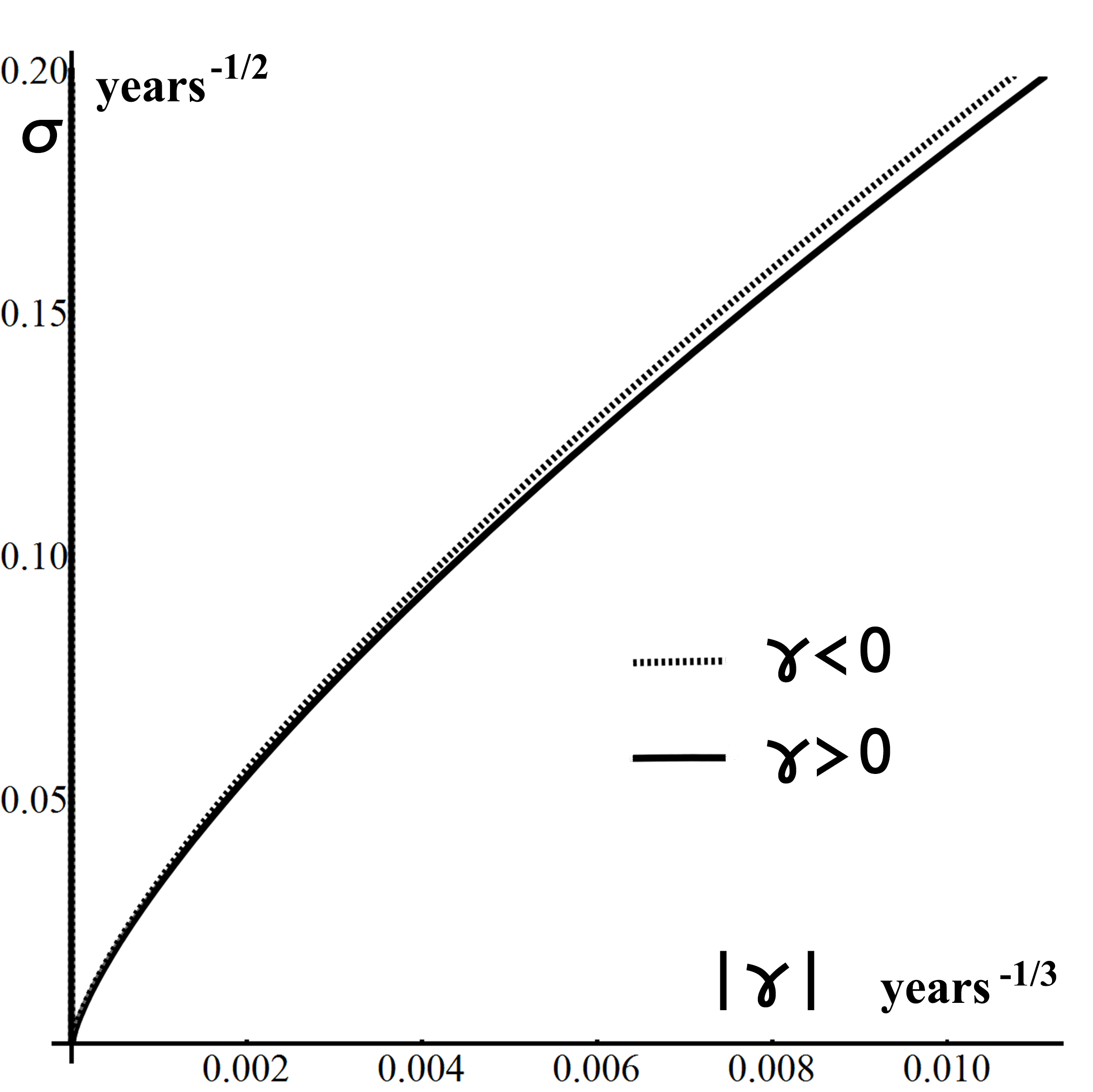

Supplement: S3 Fig — The region above the curves shows combinations of (σ, γ) for which η > η c and M min < M c for τ = 14 days, R c = 0.1, η c = 10, M c = 1/100 and r = 0.02 years−1. (TIF) [file pone.0116752.s004.tif]
